# The Effect of Nonsurgical Periodontal Therapy on *Trichomonas Tenax* and *Entamoeba Gingivalis *in Patients with Chronic Periodontitis

**Published:** 2016-09

**Authors:** Fahimeh Rashidi Maybodi, Ahmad Haerian Ardakani, Ali Fattahi Bafghi, Alireza Haerian Ardakani, Akram Zafarbakhsh

**Affiliations:** 1 Dept. of Periodontology, Shahid Sadoughi University of Medical Sciences, Yazd, Iran.; 2 Dept. of Parasitology & Mycology, Shahid Sadoughi University of Medical Sciences, Yazd, Iran.; 3 Dept. of Orthodontics, Shahid Sadoughi University of Medical Sciences, Yazd, Iran.; 4 Dept. of Periodontology, Shahrekord University of Medical Sciences, Shahrekord, Iran.

**Keywords:** Trichomonas Tenax, Entamoeba Gingivalis, Periodontitis, Parasite

## Abstract

**Statement of the Problem:**

*Trichomonas tenax* and *Entamoeba gingivalis* are commensal protozoa which inhabit the human oral cavity. These parasites are found in patients with poor oral hygiene and might be a reason for progressive periodontal diseases.

**Purpose:**

The aim of this study was to evaluate the effect of nonsurgical periodontal treatment on the frequency of these protozoa in saliva and plaque samples.

**Materials and Method:**

In this clinical trial, samples of saliva and dental plaque were collected from 46 patients with moderate to severe chronic periodontitis before and after periodontal therapy. The samples were assessed for the frequency of parasites.

**Results:**

The frequency of *Entamoeba gingivalis* was reduced in saliva (*p*= 0.007) and plaque (*p*= 0.027) three weeks after the treatment. Likewise, the frequency of *Trichomonas tenax* reduced in saliva (*p*= 0.030); however, the decrease was not significant in plaque (*p*= 0.913). Trichomonas tenax frequency in dental plaque directly related to the severity of periodontitis (r= 0.565, *p*≤ 0.000). In contrast, the number of *Entamoeba gingivalis* in both saliva (r= -0.405, *p*≤ 0.005) and plaque (r= -0.304, *p*= 0.040) was inversely related with the severity of the periodontal disease.

**Conclusion:**

Nonsurgical periodontal treatment could reduce the number of *Trichomonas Tenax *and *Entamoeba gingivalis* in the oral environment of patients with chronic periodontitis.

## Introduction

Presenting in several various clinical forms, periodontitis is considered as one of the most widespread oral diseases. Approximately 5 to 20 percent of the world’s population suffers from severe generalized periodontitis.[1] Periodontal lesions contain numerous neutrophils, motile bacteria, spirillae, spinning rods, and protozoa.[2]Yet, the etiology of this multi-factorial disease is still unclear. The possible role of parasites in the development of periodontitis has been poorly studied.[3] Data from previous studies about Entamoeba gingivalis and Trichomonas is also limited[4] and have been conducted only in few countries.[5]******

Trichomonas are mostly parasitic or commensal flagellates that live in low-oxygen environments.[6-8] *T. tenax* is currently considered as a member of the oral biofilm.[9-10] Its prevalence in the oral cavity ranges from 4 to 53% worldwide;[11] however, in patients with periodontitis; it is 3 to 4 times more than healthy individuals.[12] Investigations have been performed on the correlation between the prevalence of *T. tenax* and the status of periodontitis,[6, 13] which is found particularly in patients with poor oral hygiene.[13-15] It can be simply transmitted through saliva, droplet spray, kissing, using contaminated dishes, and drinking water.[4, 7, 11] Oral cavity is the common residence of Trichomonas species; although, it is occasionally found in the respiratory tract.[16-18] The significance of its presence in the respiratory tract of humans is still unclear. Pleuropulmonary trichomoniasis has been observed in bare cases.[19-21] A study in Egypt (2004) showed that the prevalence of pulmonary trichomoniasis was 8% (20 cases) in a total of 250 individuals.[22]

*Entamoeba gingivalis (E. gingivalis)* is found in the oropharynx, but rarely in the head and neck lesions.[23-25] This microorganism is found more common in patients with poor dentition,[24] periodontal disease, and immune suppression.[26-28] It was the first commensal found in the human oral cavity.[12, 29] *E. gingivalis* lives on the surface of the teeth and gum tissues.[30] Inflammatory process produces a propitious anaerobic environment for their growth.[31] The trophozoite is measured about 10-30 µm, actively motile with multiple pseudopodia, the cytoplasm contains food vacuoles with ingested bacteria, leukocytes and epithelial cells.[29] According to some studies, this amoeba is considered as an important cause of periodontal disease.[32] *E. gingivalis* is an opportunistic pathogen whose association with synergistic symbiotic bacteria may cause periodontal diseases in hosts with low immunity.[33-34]

Conversely, some studies have reported the general presence of *E. gingivalis* in disease-free individuals which was related to the amount of calculus present on the teeth.[35] As previously mentioned in several studies, these parasites exist in the oral cavity and probably play a role in development of periodontal disease. Yet, no study has investigated the patients with moderate to severe chronic periodontitis. Hence, the current study was designed to evaluate the effect of nonsurgical periodontal treatment on the level of *Trichomonas tenax* and *Entamoeba gingivalis* in dental plaques and saliva.

## Materials and Method

In this randomized clinical trial (#IRCT2014071013167 N4), 46 patients with moderate to severe chronic periodontitis (according to the Periodontology Association in 1998) and the age range of 30-50 years old were selected out of those referring to the Department of Periodontics, Shiraz School of Dentistry, Iran, from March to October 2014. Informed consent was collected from the patients. The participants were screened and examined to make sure if they met the criteria for periodontal disease. The exclusion criteria were having a history of consumption of systemic antibiotics within the three preceding months, periodontal therapy during the previous six months, medications that affect the periodontium (such as immunosuppressives, antihypertensives, anticonvulsants, contraceptives), systemic diseases such as diabetes, heart disease or respiratory diseases, pregnancy, smoking or other drug abuse. A demographic form was filled out for each individual concerning age, sex, smoking habits, and medical status.

As previously mentioned, the aim of this study was to evaluate the effect of nonsurgical periodontal treatment on the frequency of *T. tenax* and *E. gingivalis* in saliva and plaque samples[5, 7, 35] before and 3 weeks after intervention. The first follow-up should be considered at least 3 weeks after treatment. This interval is needed for tissue healing.[36] To this end, the patients were randomly divided into the case (23 patients) and control (23 patients) groups by a table of random numbers. Considering the severity of periodontal disease by evaluating the mean clinical attachment loss (CAL) in the patients’ periodontium, they were also divided into one of the two subgroups of moderate (CAL=3-4mm) and severe periodontitis (CAL≥5mm).[36] Sampling was done in two sessions (baseline and three week after the treatment). After collecting the first samples from all patients, the control group received only oral hygiene instructions. Scaling and root planning was done in case group via ultrasonic set (Woodpecker; China) for 20 to 60 minutes until eliminating deposits completely. Then face-to-face oral hygiene instructions were given by using a dental model. Three weeks later, the second set of samples were prepared and delivered to the lab. The examiner who studied the saliva and plaque samples was blind to the groupings throughout the study.

**Figure 1:**
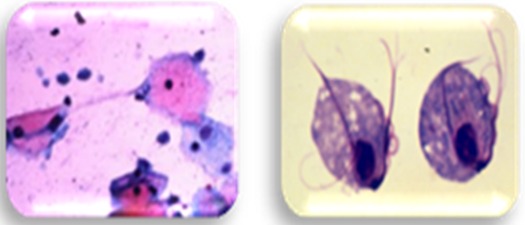
Microscopic view of *Trichomonas gingivalis*

**Figure 2:**
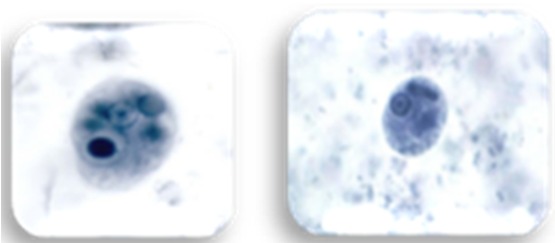
Microscopic view of *Entamoeba gingivalis*

Dental plaque was collected from the teeth surfaces by using sterile curettes (Gracey curette #13-14). Non-stimulated saliva (0.1 ml) was collected by using sterile pipettes (Hirschmann graduated pipette; Class AS, 10 mL volume, accuracy: 0.05 mL, Germany). The labeled smears of saliva and dental plaques were fixed with methanol and sent to the parasitology lab. Then, these Giemsa-stained smears were observed by using microscope (Nikon; Eclipse E200, Japan) at both 40X and 100X magnifications. Detection of *T. tenax* was established as a pear-shaped flagellated trophozoite of about 5-13µ (Figure 1). The other oral protozoan, *E. gingivalis* was differentiated by its size (10-20μ), presence of prominent pseudopodia and food vacuole (Figure 2).

Microscopic observation was repeated twice to confirm the diagnosis and distinguishing the specific pattern of each parasite family. In the microscopic study of parasites, the presence of very few number of parasites was considered as level 1 (0-1 parasites), few number as level 2 (2-4 parasites), moderate as level 3 (5-10 parasites), high as level 4 (11-15 parasites), and very high (16≤parasites) as level 5 (the results were reported qualitatively rather than quantitatively).

In this study, the probable correlation between demographic data such as age, sex, and education level and the initial abundance of parasites were analyzed in oral samples. Furthermore, the abundance of parasites after periodontal treatment was compared with the baseline values.

The statistical analysis was done by using SPSS software, version 16. The independent-samples t-test was used for variables with abnormal distribution (significance level ≤0.05). Pearson Correlation test was employed to analyze the relationship of severity of the disease (on the basis of CAL levels), age and sex with the abundance of parasites (signification level≤0.005). The changes in parasites abundance before and after the treatment were determined by using Mann-Whitney test. 

## Results

Out of all the 46 patients, 33 were females and 13 were males. There were 29 patients with severe periodontitis (15 cases and 14 controls), and 17 with moderate periodontitis (8 cases and 9 controls). The mean baseline CAL in the two groups was almost equal (case group= 5.74±1.711 mm, and control group= 5.64±1.677 mm).

Generally, the frequency of *T. tenax* was less than *E. gingivalis* in both saliva and dental plaque (*p*≤ 0.005). The severity of periodontitis was directly related with the baseline frequency of *T. tenax* in dental plaque (r= 0.565, *p*≤ 0.000); however, it had no relationship with the frequency of this parasite in saliva. The frequency of *E. gingivalis* in saliva (r=- 0.405, *p*≤ 0.05) and dental plaque (r= -0.304, *p*= 0.040) had a significantly reverse relationship with the severity of periodontal disease.

Performing the test of homogeneity of variances of parasites showed that the frequency of *T. tenax* in saliva (*p*=0.039) and dental plaque (*p*= 0.017) was statistically different in patients with different education levels. Parasites were mostly found in no-education (illiterate) group.

Post-hoc test revealed no correlation between the patient’s age and the frequency of *T. tenax* in dental plaque (*p*= 0.066) or saliva (*p*= 0.34). *T. tenax* in dental plaque was detected only in females (*p*≤ 0.005).

The frequency of *E. gingivalis* in dental plaque (*p*= 0.212) and saliva (*p*= 0.441) had no significant difference between various groups with different levels of education. It was found more frequently in males’ saliva rather than females’ (*p*≤ 0.005). No relationship was noticed between the age and colonization of *E. gingivalis* in saliva (*p*= 0.13) and dental plaque (*p*= 0.314).

Three weeks after the treatment, *E. gingivalis* decreased in the in case group's saliva (*p*= 0.007) and plaque samples (*p*= 0.027). *T. tenax* also reduced in saliva (*p*= 0.030); nevertheless, it did not have a significant reduction in dental plaque (*p*= 0.913).

## Discussion

The initial treatment to eliminate pathogenic bacteria in periodontal disease is the mechanical removal of dental plaque and calculus.[37-39] Consequently, it was supposed that these methods could help reduce parasites as much as it does for perio-pathogen bacteria. The obtained results confirmed the probable efficacy of periodontal treatment in reducing the colonization of oral parasites in dental plaque except for *T.tenax*. No similar study was found about the effect of periodontal treatment on the management of oral parasites; however, some studies have evaluated the prevalence of these protozoans.

Ghabanchi *et al.,*[7] observed that *E. gingivalis* was more abundant than *T. tenax* in patients with either healthy periodontium or periodontitis, which was consistent with the results of the present study. In a study conducted by Bonner *et al.*, 72 periodontal pockets and 33 healthy gingival sulci were sampled to evaluate the correlation between *E. gingivalis* colonization and periodontitis by using PCR (polymerase chain reaction).[3] Although Bonner used a much more sensitive technique (PCR) compared with this study (light microscope), both studies noted higher frequency of *E. gingivalis* compared with *T.tenax*. The only difference was that in our study, there was a significant reverse correlation between the periodontal disease and *E. gingivalis* frequency in saliva. Meanwhile, Bonner *et al.* stated that the presence of parasite was directly related to the periodontal disease in an overall view. Ibrahim and Abbas reported a direct relationship between the severity of periodontitis and abundance of *E. gingivalis* and *T. tenax* in both saliva and plaque.[26] The current study detected a direct correlation between the frequency of *T. tenax* in plaque and severity of periodontal disease; whereas, this direct relationship was not observed in salivary *T. tenax* or generally in *E. gingivalis*. It should be noted that the level of CAL was not considered as a determining factor in the severity of periodontal disease in Ibrahim’s study,[26] only the pocket depth was measured. Hence, the relationship between the parasites and the disease severity cannot be confirmed with certainty. They also reported a higher abundance of *E. gingivalis* in hypertensive patients and *T. tenax* in patients with increased use of antibiotics over the preceding 6 months. Unlike the present study, they did not exclude patients with systemic complications and found that both parasites increased in diabetic patients.

Ibrahim and Abbas observed that *E. gingivalis* was more frequent in 61-70 year-old and *T. tenax* in 21-30 year-old patients,[26] while; the age parameter had no significance in our study. Ghabanchi *et al.* detected that *E. gingivalis* could be found in dental plaque and saliva of healthy subjects.[7] Our study showed a reverse significant correlation between the severity of periodontitis and *E. gingivalis* frequency, which may confirm the abundance of this parasite even in healthy conditions. In contrast, Trim *et al.*[35] reported that *E. gingivalis* was only present in pockets deeper than 4-7 mm and this result was confirmed by Albuquerque *et al.*[12] and contrasted by the current study. This may be due to the differences in laboratory procedures. Hamad *et al.*[40] showed a positive relationship between the presence of parasite in the mouth and illiteracy or low education level which was partially confirmed by the present study. In our study, *T. tenax* was mostly found in illiterate patients’ saliva or dental plaque, but there was no relationship between the amount of *E. gingivalis* and various education levels. In a study, Ullah *et al.*[41] reported a positive association between the prevalence of mouth parasites and poverty. The higher incidence was found among the lower class which was to some extent in line with our study. The above-mentioned study also reported that the overall prevalence of these two parasites was higher in men than women. Our study found *T. tenax* only in females’ dental plaque. This might be attributed to the higher prevalence of *T. vaginalis* in females and the microscopic similarities of this species and *T. tenax*.[6] *E. gingivalis* was more prevalent in males’ saliva than females’. Ullah *et al.*[41] included smokers, snuff dippers and participants with improper brushing habits or poor oral hygiene in their study and as expected, the presence of these confounding factors was more frequent in males rather than females.

Gharavi *et al.*[5] found no relationship between *T. tenax* infection and sex or age, but *E. gingivalis* was related to age higher than 20 and male sex. Contrarily, Albuquerque *et al.* reported no correlation between age and the presence of both parasites, which was in agreement with the results of the present study.

## Conclusion

Regardless of patient’s demographic characteristics, it seems that oral hygiene instructions in combination with scaling and root planning can help controlling excessive colonization of parasites, particularly *E. gingivalis* and *T. tenax* and their probable opportunistic infestation.
